# Characterization of a complete mitochondrial genome of *Turdus ruficollis* (Passeriformes: Turdidae)

**DOI:** 10.1080/23802359.2021.1926359

**Published:** 2021-05-13

**Authors:** Xuze Zhang, Xiaodong Ma, Jinqiang Ha, Yingting Wei, Ying Zhao, Shengnan Wei, Xiaojie Zhong, Zhenyuan Cai

**Affiliations:** a College of Ecological Environment and Resources, Qinghai Nationalities University, Xining, China; b Institute of Ecological Environment, Qinghai Nationalities University, Xining, China; c Qinghai Provincial Key Laboratory of Animal Ecological Genomics, Xining, China; d Qinghai National Park Bird Watching Society, Xining, China; e Northwest Institute of Plateau Biology, Chinese Academy of Sciences, Xining, China

**Keywords:** *Turdus ruficollis*, mitochondrial genome, phylogenetic relationship

## Abstract

*Turdus ruficollis* is mainly found in China and Northeast Asia. So far, the mitochondrial genome of more than 20 species from the genus *Turdus* has been studied. However, the relevant information of *T. ruficollis* has not been reported. To grasp a better comprehension on the molecular basis of *T. ruficollis*, we obtained the complete mitochondrial DNA genome sequences of this species. The mitogenome was 16,737 bp in length, which consists of 13 protein-coding genes, 22 tRNA genes, two rRNA genes, and one control region. A phylogenetic tree based on complete mitogenome sequences revealed that, within the genus *Turdidae*, *T. ruficollis* is closely related to *T. naumanni* and *T. eumomus*. The complete mitochondrial genome of *T. ruficollis* would be of great utility for population genetics and phylogeography of the Turdidae family and would also provide meritorious insights for future studies on conservation, genetics, and phylogeny of the Passeriformes family.

*Turdus ruficollis* (Passeriformes: Turdidae), known as red-necked thrush, breeds in South-Central Siberia and winters in northern China (Liu et al. 1987; Xu et al. 2010; Zheng 2017). In this study, the sample was taken from a red-necked thrush with unknown cause of death on campus of Qinghai Nationalities University, Xining City, Qinghai Province, China (36.5874N; 101.8199E). The specimen of *T. ruficollis* was deposited at the animal specimen room in Herbarium of College of Ecological Environment and Resources, Qinghai Nationalities University (www.qhmu.edu.cn, Zhang Xuze, zxz1904@126.com) under the voucher number XNTR-01. Genomic DNA was extracted from the muscle tissue, using a modified method adopted from the standard phenol/chloroform extraction process (Sambrook et al. 1989), with digestion time extended to 12 hours and an additional round of phenol/chloroform extraction.

The DNA extract was sheared and sequenced on an Illumina NovaSeq (Illumina, San Diego, CA) following standard Illumina pair-end sequencing protocol with a read length of 150 bp. The average coverage of genome was about 5627. Raw reads were filtered with fastp (version 0.20.0, https://github.com/OpenGene/fastp) and 22,284,470 clean pair-end reads were assembled into a complete mitogenome with SPAdes (Bankevich et al. 2012). The whole sequence was annotated with the software Geneious v11.1.5, and tRNA genes were predicted by the online software MITOS (Bernt et al. 2013; Zhang et al. 2020).

The assembled *T. ruficollis* mitogenome was a closed loop of 16,737 bp, of which 11,396 nucleotides were coding DNA. The mitochondrial DNA sequence has been deposited in GenBank (accession no. MT712159). The mitogenome comprised 13 protein-coding genes (PCGs), 22 tRNAs, two rRNAs, and one control region. The overall base composition of the whole mitochondrial genome was 29.28% A, 23.27% T, 32.5% C, and 14.95% G, exhibiting obvious AT bias (52.55%), consistent with the typical base bias found in Turdidae mitochondria (Li et al. 2014; Sun et al. 2020).

Phylogenetic relationship of *T. ruficollis* with 18 species of Passeriformes base on the complete sequences of mitochondrial DNA was resolved by means of maximum-likelihood (ML). The ML tree was built with MEGA X (Kumar et al. 2018), based on the Kimura 2-parameter model, with bootstrap set to 1000 and *Gracula religiosa* taken as an outgroup. The phylogenetic tree suggested that *T. ruficollis* and (*T. naumanni* + *T. eumomus*) were clustered into one branch with high nodal support (100) (Figure 1). The species from the Turdidae family and those from the Muscicapidae family clustered into two separate branches. The present study has confirmed the consistency between morphological classification and molecular classification (Cheng 1987; Zheng 2017; Dong et al. 2018). The sequencing of the red-necked thrush mitochondrial genome is of particular interest for better understanding of population genetics and phylogeography of the Turdidae family. This study would also provide more basic data on conservation, genetics and phylogeny of the Passeriformes family for future research.

**Figure 1.**
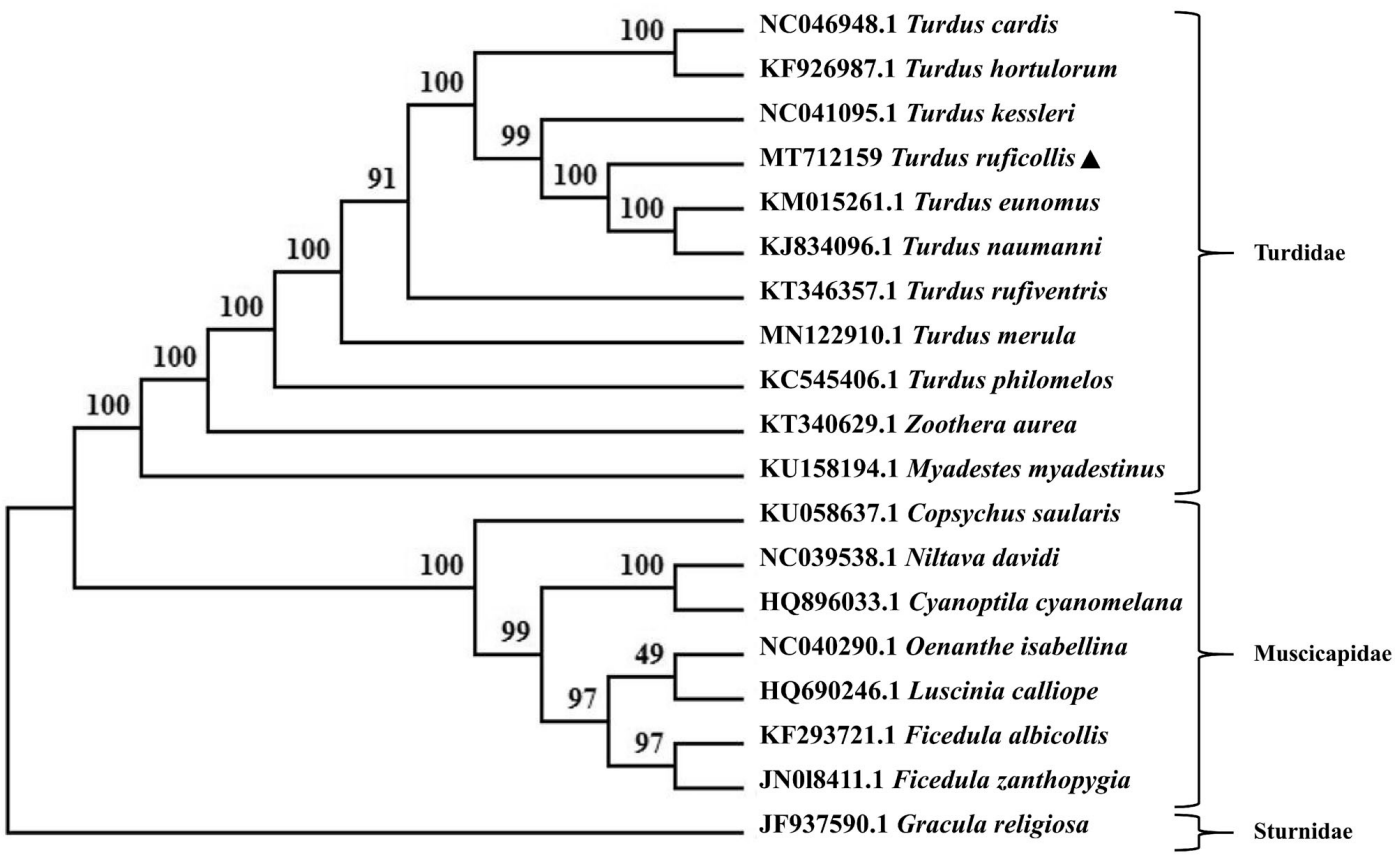
The maximum-likelihood tree based on 18 mitochondrial DNA sequences, with an outgroup *Gracula religiosa*.

## Data Availability

The mitochondrial genome sequence in the present study is openly available at GenBank (https://www.ncbi.nlm.nih.gov/) under the accession number MT712159. The associated BioProject, SRA, and Bio-Sample numbers are PRJNA679709, SUB8581141, and SAMN16844544, respectively.
